# The Acquisition of the *scr* Gene Cluster Encoding Sucrose Metabolization Enzymes Enables Strains of *Vibrio parahaemolyticus* and *Vibrio vulnificus* to Utilize Sucrose as Carbon Source

**DOI:** 10.3389/fmicb.2021.754464

**Published:** 2021-11-15

**Authors:** Jens Andre Hammerl, Cornelia Göllner, Claudia Jäckel, Fatima Swidan, Helena Gutmann, Eckhard Strauch

**Affiliations:** Department Biological Safety, German Federal Institute for Risk Assessment, Berlin, Germany

**Keywords:** *Vibrio parahaemolyticus*, metabolic activity, cloning, shuttle vector pVv3, whole-genome sequencing

## Abstract

Most strains of *Vibrio parahaemolyticus* are unable to utilize sucrose as carbon source, though few exceptions exist. We investigated a sucrose-positive *V. parahaemolyticus* strain by whole-genome sequencing (WGS) and confirmed the presences of a genomic island containing sucrose utilization genes. A 4.7 kb DNA cluster consisting of three genes: *scrA* encoding a sucrose uptake protein, *scrK* encoding a fructokinase, and *scrB* coding for a sucrose-6-phosphate hydrolase, was PCR amplified and inserted into the *Vibrio/Escherichia coli* shuttle vector pVv3. Two recombinant plasmids, only differing in the orientation of the insert with respect to the pVv3-*lacZ*α-fragment, conferred the *E. coli* K12 transformants the ability to utilize sucrose. The introduction of the two plasmids into sucrose-negative *V. parahaemolyticus* and *V. vulnificus* strains also results in a change of the sucrose utilization phenotype from negative to positive. By performing a multiplex PCR targeting *scrA, scrK*, and *scrB*, 43 *scr*-positive *V. parahaemolyticus* isolates from our collection of retail strains were detected and confirmed to be able to use sucrose as carbon source. Strains unable to utilize the disaccharide were negative by PCR for the *scr* genes. For *in-depth* characterization, 17 sucrose-positive *V. parahaemolyticus* were subjected to WGS. A genomic island with a nucleotide identity of >95% containing *scrA, scrB, scrK* and three additional coding sequences (CDS) were identified in all strains. The additional genes were predicted as a gene coding for a transcriptional regulator (*scrR*), a porin encoding gene and a CDS of unknown function. Sequence comparison indicated that the genomic island was located in the same region of the chromosome II in all analyzed *V. parahaemolyticus* strains. Structural comparison of the genomes with sequences of the sucrose utilizing species *V. alginolyticus* revealed the same genomic island, which indicates a possible distribution of this genetic structure by horizontal gene transfer. The comparison of all genome sequences based on SNP differences reveals that the presence of sucrose utilizing genes is found in genetically diverse *V. parahaemolyticus* strains and is not restricted to a subset of closely related strains.

## Introduction

*Vibrionaceae* are halophilic gram-negative bacteria and natural inhabitants of marine environments worldwide. Strains of *Vibrio* (*V.*) *parahaemolyticus* and *V. vulnificus* can cause gastrointestinal infections in humans through consumption of raw or undercooked seafood. *V. vulnificus* is also a notorious pathogen as it can cause wound infections with a fatal outcome (Baker-Austin et al., 2018). *Vibrionaceae* are present in a variety of aquatic environments and can be found in the water column, sediments or in association with other organisms (i.e., algae, fish, seafood). Due to the diversity of habitats *Vibrio* species are able to grow in a wide range of temperatures from 4 to 37°C (Farmer et al., 2015). They are facultative anaerobes capable of metabolizing many carbohydrates under aerobic or non-aerobic conditions. Differences in the ability to utilize sugars have been used as a general criterion for their biochemical identification and differentiation for a long time. Metabolization of the disaccharide sucrose is an important phenotype in the primary routine diagnostic of *Vibrio* species. Thiosulfate-Citrate-Bile-Sucrose (TCBS) agar is commonly used as first medium for the isolation of vibrios from different sources (Farmer et al., 2015; Hartnell et al., 2019). Colonies of *Vibrio* bacteria that utilize sucrose appear yellow on TCBS agar, while those unable to metabolize this sugar are green/blueish. Determination of sucrose utilization is also part in traditional biochemical testing series for species identification. Many food laboratories still use the biochemical identification of *Vibrio* isolates obtained from various seafood sources, as *V. parahaemolyticus* is a major cause of gastrointestinal infections worldwide (DIN EN ISO 21872-1:2017-10) (ISO 21872-1:2017-10, 2017).

Most strains of the species *V. vulnificus* and *V. parahaemolyticus* are unable to use sucrose as carbon source, although exceptions occur. While up to 15% of *V. vulnificus* strains are sucrose-positive, only ∼1% of the *V. parahaemolyticus* isolates were reported to ferment sucrose (Lam, 1985; Farmer et al., 2015). All strains of *V. alginolyticus*, a species frequently co-isolated with *V. parahaemolyticus*, are able to ferment sucrose. Thus, sucrose utilization represents an important phenotype for the differentiation of both species. Nowadays, commonly molecular techniques, like PCR, DNA sequencing or MALDI-ToF mass spectrometry are used for rapid and reliable species identification (Bauer and Roervik, 2007; Dieckmann et al., 2010). However, a sucrose-positive phenotype will not be detected by using these techniques in the current routine diagnostic.

Sucrose uptake and degradation in *V. alginolyticus* follow a pathway that is common for carbohydrate utilization in many eubacteria. Sucrose is taken up and translocated into the bacterial cell by phosphorylation through a sugar specific enzyme of the phosphenolpyruvate (PEP):carbohydrate phosphotransferase system (PTS) (Postma et al., 1993; Deutscher et al., 2006). Inside the bacterial cell sucrose-6-phosphate is cleaved by hydrolysis of the glycosidic bond. The two resulting monosaccharides glucose-6-phosphate and fructose enter the glycolytic pathway directly or in case of fructose are phosphorylated first (Reid and Abratt, 2005). The genetics of sucrose uptake and degradation of *V. alginolyticus* was characterized and all required genes were found organized in a cluster (Scholle et al., 1987, 1989; Blatch et al., 1990; Blatch and Woods, 1991). The cluster contains the gene for a sugar specific PTS gene (*scrA*) for uptake of the disaccharide. Downstream of *scrA*, a gene for fructokinase (*scrK*) and a gene encoding sucrose-6-phosphate hydrolase (*scrB*) are located. Upstream of *scrA* a gene encoding a regulator (*scrR*) is found whose transcription is in the opposite direction to *scrA, scrK*, and *scrB*. In one recent paper the genetics of sucrose metabolism in several *Vibrio* and *Photobacterium* species was addressed (Abushattal et al., 2020) and in a second paper the genes of a sucrose-positive *V. parahaemolyticus* isolate were identified (De Mesa et al., 2021). The arrangement of sucrose utilization genes of most species correspond to that of *V. alginolyticus*.

In the past, we obtained a number of sucrose-positive *V. parahaemolyticus* strains from different food sources. To elucidate the genetic background enabling sucrose utilization of these isolates, we performed whole-genome sequencing (WGS) and bioinformatics analysis of one isolate. We also identified a gene cluster (abbreviated *scr* gene cluster) which showed high similarity to the *V. alginolyticus* cluster. We cloned the corresponding region of genes responsible for translocation and metabolization of sucrose into a *Vibrio/Escherichia coli* shuttle cloning vector. Resulting recombinant plasmids were introduced into sucrose-negative strains of *V. vulnificus* and *V. parahaemolyticus* to analyze the sucrose phenotype of the transformants. A multiplex PCR targeting the individual determinants of the *scr* gene cluster was developed to elucidate their genetic dissection in other *scr*-positive *V. parahaemolyticus* isolates. WGS analyses on more sucrose-positive strains were performed to determine the genetic arrangement in all sequenced strains and to find out if sucrose utilization is restricted to phylogenetically closely related strains.

## Materials and Methods

### Bacterial Strains, Culture Conditions, and Initial Identification

Bacterial strains used for cloning and expression of the *scr* genes are displayed in Table 1. Information regarding the source and time of isolation of *Vibrio* strains are shown in Table 2. Most *V. parahaemolyticus* strains were sent to the *Vibrio* consultant laboratory hosted at the BfR from official German federal state office laboratories, which investigate seafood for microbiological safety. All strains used in this study were routinely grown on lysogeny broth (LB) containing yeast extract (5 g/L), peptone (10 g/L), NaCl (10 g/L) or LB agar (Merck, Darmstadt, Germany). For selective cultivation of strains carrying the shuttle vector pVv3 (Klevanskaa et al., 2014) or its derivatives, kanamycin sulfate (Roth, Karlsruhe, Germany) was supplemented to a final concentration of 100 μg/mL (w/v).

**TABLE 1:** Strains used for cloning and expression of *scr* operon.

Species^a^	Strain ID	Source	Sucrose utilization
*E. coli* K12	GeneHogs^®^	Invitrogen (Thermo Fisher)	Negative
*E. coli* K12 [pVv3]^b^	GeneHogs^®^	Invitrogen (Thermo Fisher)	Negative
*V. parahaemolyticus*	16-VB00198	Retail strain	Positive
*V. parahaemolyticus*	BfR-VB00009	Reference strain WDCM00037	Negative
*V. parahaemolyticus*	BfR-VB00016	Reference strain WDCM00185	Negative
*V. vulnificus*	BfR-VB00034	Reference strain WDCM00187	Negative

*^a^See Table 2 for more information on Vibrio strains.*

*^b^pVv3: shuttle vector with broad host range for Vibrio spp. and multiple cloning site in lacZα fragment (Klevanskaa et al., 2014).*

**TABLE 2:** Source, isolation year and country of *Vibrio* strains used in this study.

Species	Strain-ID	Source	Year of isolation	Country
*V. parahaemolyticus* ^*a*^	BfR-VB00009	Reference strain – WDCM00037	Before 1995	Japan
*V. parahaemolyticus* ^*a*^	BfR-VB00016	Reference strain – WDCM00185	1972	United Kingdom
*V. vulnificus* ^*a*^	BfR-VB00034	Reference strain – WDCM00187	1976	United States
*V. parahaemolyticus*	16-VB00019	*Penaeus* shrimps	2016	Retail Germany^c^
*V. parahaemolyticus*	16-VB00101	*Penaeus* shrimps	2016	Retail Germany^c^
*V. parahaemolyticus*	16-VB00133	*Penaeus* shrimps, frozen	2016	Ecuador
*V. parahaemolyticus*	16-VB00138	Common shrimp (*Crangon* sp.)	2016	Germany, North Sea
*V. parahaemolyticus*	16-VB00150	Common shrimp (*Crangon* sp.)	2016	Germany, North Sea
*V. parahaemolyticus*	16-VB00151	Common shrimp (*Crangon* sp.)	2016	Germany, North Sea
*V. parahaemolyticus*	16-VB00152	Common shrimp (*Crangon* sp.)	2016	Germany, North Sea
*V. parahaemolyticus* ^*b*^	16-VB00179	Common shrimp (*Crangon* sp.)	2016	Germany, North Sea
*V. parahaemolyticus* ^*b*^	16-VB00184	*Penaeus* shrimps, raw with shell	2016	Vietnam
*V. parahaemolyticus*	16-VB00189	*Penaeus* shrimps, raw with shell	2016	Vietnam
*V. parahaemolyticus*	16-VB00196	*Penaeus* shrimps, skewers	2016	Retail Germany^c^
*V. parahaemolyticus* ^*b*^	16-VB00198	Oyster (*Crassostrea gigas*)	2016	Germany, North Sea
*V. parahaemolyticus* ^*b*^	16-VB00204	Blue mussel (*Mytilus edulis*)	2016	Northeast atlantic
*V. parahaemolyticus*	16-VB00215	Giant Tiger Prawn (*Penaeus monodon*)	2016	India
*V. parahaemolyticus*	16-VB00220	*Penaeus* shrimps, raw with shell	2016	Thailand
*V. parahaemolyticus*	17-VB00043	Whiteleg shrimp (*Penaeus vannamei*)	2017	India
*V. parahaemolyticus* ^*b*^	17-VB00065	Blue mussel (*Mytilus edulis*)	2017	Germany, North Sea
*V. parahaemolyticus*	17-VB00069	*Penaeus* shrimps, frozen	2017	Ecuador
*V. parahaemolyticus*	17-VB00071	*Penaeus* shrimps	2017	Ecuador
*V. parahaemolyticus*	17-VB00072	*Penaeus* shrimps	2017	Ecuador
*V. parahaemolyticus*	17-VB00086	*Penaeus* shrimps	2017	Ecuador
*V. parahaemolyticus*	17-VB00093	*Penaeus* shrimps	2017	Ecuador
*V. parahaemolyticus*	17-VB00100	*Penaeus* shrimps	2017	Ecuador
*V. parahaemolyticus*	17-VB00108	*Penaeus* shrimps	2017	Ecuador
*V. parahaemolyticus* ^*b*^	17-VB00127	*Penaeus* shrimps	2017	Vietnam
*V. parahaemolyticus* ^*b*^	17-VB00129	*Penaeus* shrimps	2017	Thailand
*V. parahaemolyticus* ^*b*^	17-VB00146	Whiteleg shrimp (*Penaeus vannamei*)	2017	Vietnam
*V. parahaemolyticus*	17-VB00157	Blue mussel (*Mytilus edulis*)	2017	Germany, North Sea
*V. parahaemolyticus*	17-VB00160	Blue mussel (*Mytilus edulis*)	2017	Germany, North Sea
*V. parahaemolyticus* ^*b*^	17-VB00161	Blue mussel (*Mytilus edulis*)	2017	Germany, North Sea
*V. parahaemolyticus* ^*b*^	17-VB00208	*Penaeus* shrimps	2017	India
*V. parahaemolyticus* ^*b*^	17-VB00250	Common shrimp (*Crangon* sp.)	2017	Germany, North Sea
*V. parahaemolyticus*	17-VB00256	Common shrimp (*Crangon* sp.)	2017	Germany, North Sea
*V. parahaemolyticus*	17-VB00276	*Penaeus* shrimps	2017	Ecuador
*V. parahaemolyticus*	17-VB00287	Blue mussel (*Mytilus edulis*)	2017	Germany, North Sea
*V. parahaemolyticus*	17-VB00300	Common shrimp (*Crangon* sp.)	2017	Germany, North Sea
*V. parahaemolyticus* ^*b*^	17-VB00303	Common shrimp (*Crangon* sp.)	2017	Germany, North Sea
*V. parahaemolyticus* ^*b*^	17-VB00319	Common shrimp (*Crangon* sp.)	2017	Germany, North Sea
*V. parahaemolyticus*	17-VB00323	Common shrimp (*Crangon* sp.)	2017	Germany, North Sea
*V. parahaemolyticus* ^*b*^	17-VB00330	Common shrimp (*Crangon* sp.)	2017	Germany, North Sea
*V. parahaemolyticus* ^*b*^	17-VB00358	*Penaeus* shrimps	2017	Honduras
*V. parahaemolyticus* ^*b*^	17-VB00364	*Penaeus* shrimps	2017	Vietnam
*V. parahaemolyticus* ^*b*^	17-VB00366	*Penaeus* shrimps	2017	Ecuador

*^a^Strains negative for sucrose utilization.*

*^b^Strains used for whole genome sequencing.*

*^c^No information on geographical origin.*

Identification of *V. parahaemolyticus* strains was carried out using phenotypical tests, e.g., cultivation on TCBS agar (Oxoid, Wesel, Germany) and *Vibrio* Chrom-Agar (MAST-Diagnostika, Reinfeld, Germany), growth tolerance against different NaCl concentrations (0, 2, 6, 8, and 10%) in peptone water (Bacto Peptone, Gibco/Thermo Fisher Scientific, Schwerte, Germany; NaCl Merck, Darmstadt, Germany), API20E profiling (biomérieux, Nürtingen, Germany) and oxidase test (Merck, Darmstadt, Germany). Furthermore, PCR targeting the *toxR* gene (Bauer and Roervik, 2007; Bechlars et al., 2015) and Matrix-Assisted-Laser-Desorption/Ionization Time-of-Flight Mass Spectrometry (MALDI-ToF MS) (Biotyper microflex, LT/SH, Bruker, Bremen, Germany) was conducted as previously described (Jäckel et al., 2020). The test of sucrose fermentation was conducted by using sucrose bouillon with bromothymol blue containing peptone from meat (10 g/L), NaCl (5 g/L), and sucrose (10 g/L) (Merck, Darmstadt, Germany). 5 mL of sucrose broth was inoculated with bacterial strains and incubated for 24–48 h at 37°C. Sucrose-positive strains turn the media from blueish green to yellow.

### Molecular Amplification of the *scr* Genes

Genomic DNA (gDNA) extraction from *V. parahaemolyticus* strain 16-VB00198 was carried out by using the RTP Bacteria DNA Kit (Stratec Molecular, Berlin, Germany) according to the recommendations of the manufacturer and was used for multiplex PCR amplification of the *scr* gene cluster. PCR reactions for the detection of the *scrA*, *scrB*, and *scrK* genes were performed in a volume of 25 μl with 1x PCR buffer (2 mM MgCl_2_), 0.2 mM of each dNTP, 0.5 μM of each primer, and 1.5 U of Dream Taq DNA Polymerase (Fermentas, St. Leon-Rot, Germany). The following PCR running conditions were used: An initial denaturation of 94°C for 2 min, 35 cycles of 94°C for 15 s, 58°C for 30 s, and 72°C for 30 min, and a final elongation step of 72°C for 5 min. The sequences of the used oligonucleotide primers are given in Table 3.

**TABLE 3:** PCR Primers used for amplification of *scr* genes.

Target	Primer	Sequence 5′- 3′	Annealing [C°]	Product [bp]
*scrA*	VparascrA-Fo	CGG CAT TCA TTC TTG CGA AG	58	331
	VparascrA-Re	CAT CGC CGC AAT AGG AAA G		
*scrB*	VparascrB-Fo	AAC GTG GCA GCA TAA AGG TC	58	527
	VparascrB-Re	AGT TCA AAG CCA TCG CCC TC		
*scrK*	VparascrK-Fo	ACT TCT TTA CTC AAA TGT CCC G	58	410
	VparascrK-Re	AGG GTC AAA GCT AAT GAA ACC		
*scr* cluster	VparascrAB-Fo	GGA GGA TCC GTT CCC GAA AAT TTC GCA AG	61.6	4656
	VparascrAB-Re	ATG AAG CTT GGA ATG TAT GCT CTC CAA CTG		

*BamHI (-GGATCC-) and HindIII (-AAGCTT-) restriction sites are underlined.*

### Cloning of *scr* Gene Cluster

For the amplification of the complete *scr* gene cluster comprising *scrA*, *scrK*, and *scrB* and the flanking regions, the Phusion^TM^ High-Fidelity DNA Polymerase Kit (ThermoFisher Scientific, Schwerte, Germany) was used with the primers VparascrAB-Fo and VparascrAB-Re (Table 3). In a volume of 50 μl, 1 x PCR buffer (2 mM MgCl_2_), 0.2 mM of each dNTP, 0.5 μM of primers and 1.5 U of Phusion^TM^ Polymerase (Thermo Fisher Scientific, Schwerte, DE) were used for amplification. PCR conditions were applied as stated below: An initial denaturation of 98°C for 2 min, 35 cycles of 98°C for 10 s, 61.6°C for 30 s and 72°C for 2 min, and a final elongation step of 72°C for 5 min. For further downstream analysis, the PCR products were purified using the RTP Kit INVITEK according to the manufacturer’s recommendations (Stratec Molecular, Berlin, Germany). A 4,656 bp PCR fragment containing the *scr* operon was obtained from *V. parahaemolyticus* 16-VB00168. The fragment was digested with the enzymes *Bam*HI and *Hin*dIII (Thermo Fisher Scientific, Schwerte, Germany), whose recognition sequences were included in the sequences of the primers VparascrAB-Fo and VparascrAB-Re (Table 3). The digested PCR fragment was inserted into the corresponding sites of the pUC18 cloning vector (Thermo Fisher Scientific, Schwerte, Germany) using T4 ligase (Thermo Fisher Scientific, Schwerte, Germany). After transformation of electrocompetent *E. coli* GeneHogs^TM^ cells, transformants containing the recombinant plasmid consisting of pUC18 with the inserted *scr* operon were obtained on LB agar with ampicillin (Merck, Darmstadt, DE) (100 mg/mL). Plasmid DNA from transformants was isolated using the RTP plasmid Kit according to the manufacturer’s recommendation and subjected to Sanger sequencing (Eurofins Genomics, Ebersberg, Germany) for verification.

The verified *scr* region was excised from the recombinant pUC18 derivative by *Bam*HI/*Hin*dIII cleavage and inserted into the corresponding sites of the pVv3 shuttle cloning vector (accession number HG326273) (Klevanskaa et al., 2014). To avoid re-ligation of the vector DNA, *Bam*HI/*Hin*dIII cleaved pVv3 was incubated with alkaline phosphatase (Thermo Fisher Scientific, Schwerte, Germany) before T4 ligase treatment. Ligation mixtures were subjected to transformation to electrocompetent *E. coli* GeneHogs cells. Transformed bacteria were selected on LB agar supplemented with kanamycin (100 μg/mL) and the presence of the *src* gene cluster was confirmed by primer walking (Supplementary Table S1) and Sanger sequencing (Eurofins Genomics, Ebersberg, Germany). pVv3 plasmid or derivative isolation was conducted using Qiagen Plasmid Plus Kits (Qiagen GmbH, Hilden, Germany).

Preparation of competent cells of *E. coli* GeneHogs^TM^ was done following a standard protocol (Sambrook and Russel, 2001). The preparation of electrocompetent *Vibrio* cells was conducted as described previously (Klevanskaa et al., 2014). For electroporation, 200–400 ng gDNA and 40–50 μl freshly prepared competent cells were mixed, and incubated on ice for 10 min. Electroporation was performed at settings of 7.5 kV cm^–1^, 25 μF, and 200 Ω. Thereafter, 950 μl SOC medium (Sambrook and Russel, 2001) was added, and the bacteria were incubated for 37°C for 1 h. Afterward the bacteria were plated on LB agar under selective conditions, but potential *Vibrio* transformants were investigated by MALDI-ToF MS before further processing.

### Whole-Genome Sequence Determination and Bioinformatics Analysis

Whole-genome sequencing was performed for 17 *V. parahaemolyticus* isolates. Therefore, isolates were grown in LB and gDNA was extracted using the PureLink Genomic DNA Kit (Invitrogen, Karlsruhe, Germany). Paired-end, short-read WGS (MiSeq, Illumina, San Diego, CA, United States) was performed as previously described (Schwartz et al., 2019). SPAdes *de novo* assemblies using raw reads and genome annotation were performed using the Pathosystems Resource Integration Center (PATRIC) (release 3.5.39) (Wattam et al., 2017) and the automated Prokaryotic Genome Annotation Pipeline (PGAP) of the National Center for Biotechnology Information (NCBI), respectively.

Average nucleotide identity (ANI) prediction was used for assigning genomes at species level (Rodriguez and Konstantinidis, 2014). Genome sequences were compared pairwise to the genome of the *V. parahaemolyticus* type strain ATCC 17802 (accession NZ_LATW01000001-51). The ANI online calculation tool^1^ was used with default settings (alignment options: minimum length 700 bp, minimum identity 70%, minimum alignments 50; fragment options: window size 1,000 bp, step size 200 bp).

Multilocus sequence typing (MLST) was conducted using MLST Finder (Larsen et al., 2012), which is based on the *V. parahaemolyticus* scheme of the pubMLST database^2^ (Jolley et al., 2018). Prediction of putative prophage sequences was performed using the PHAge Search Tool Enhanced Release (PHASTER) with default settings (Arndt et al., 2016). Initial plasmid replicon prediction was performed using PlasmidFinder (v 2.0^3^) (Carattoli et al., 2014). In addition, genomic contigs showing significantly higher sequence coverages than the rest of the contigs were screened for similarities to known plasmids using the BlastN algorithm of the NCBI database All BlastN searches were carried out using the NCBI database (https://blast.ncbi.nlm.nih.gov/Blast.cgi?PROGRAM=blastn&PAGE_TYPE=BlastSearch&LINK_LOC=blasthome) with default settings.

To determine the phylogenetic relationship of the isolates, a CSI Phylogeny (v 1.4^4^) based single nucleotide polymorphism (SNP) tree was prepared. The tool was used under default settings and the exclusion of heterozygous SNPs. As reference for comparison of the sucrose-positive strains the WGS dataset of 16-VB00498 was used. Nucleotide variations were predicted according to the specifications provided (Kaas et al., 2014).

The relation between the sucrose region (*scrA-scrK-scrB*) was determined by using MEGA X (Kumar et al., 2018) with the Maximum Likelihood method and Tamura–Nei model (Tamura and Nei, 1993). Initial tree(s) were obtained automatically by applying Neighbor-Join and BioNJ algorithms. Bootstrapping was performed with 500 repetitions using all sites.

### Accession Numbers

Genome sequences of the investigated *V. parahaemolyticus* isolates have been deposited in GenBank at the National Center for Biotechnology Information (NCBI) under the accession numbers given in Supplementary Table S2.

## Results

### Identification of a Sucrose Utilization (*scr*) Gene Cluster in *Vibrio parahaemolyticus* 16-VB00198

16-VB00198 was assigned to the species *V. parahaemolyticus* by MALDI-ToF MS and PCR targeting *toxR* (data not shown). The strain was positive for sucrose utilization when tested with API20E strips. Furthermore, it changed the color from blue to yellow when grown in sucrose bouillon indicating that it is able to utilize sucrose as sole carbon source.

Whole-genome sequencing and ANI calculation showed a result of 98.2% identity between 16-VB00198 and the *V. parahaemolyticus* type strain ATCC17805 (WDCM00037). Through BlastN searches using published genes involved in sucrose metabolism, a CDS cluster consisting of three *scr* genes was identified. Nucleotide sequence (accession M30194) containing the sucrose uptake region with *scrA* and *scrB* genes of *V. alginolyticus* (Blatch et al., 1990) showed a nucleotide identity of 97% to a DNA region (contig_16) of 16-VB00198. Further bioinformatic analysis revealed that the strain contains a sucrose utilization gene cluster containing three genes (*scrA, scrK*, and *scrB*) arranged head to tail in the order 5′-3′. The *scrA* gene encodes a sucrose uptake protein of the phosphoenolpyruvate dependent phosphotransferase system (PTS), *scrK* encodes a fructokinase and *scrB* a sucrose-6-phosphate hydrolase (Scholle et al., 1987, 1989; Blatch and Woods, 1991; Postma et al., 1993). Upstream of *scrA* a gene coding for a transcriptional regulator is located whose coding sequence is counter orientated to *scrA*.

### Cloning of the *scrAKB* Gene Cluster in *Escherichia coli*

To examine the functionality of the *scrA-scrK-scrB* cluster, the region was amplified by PCR with a High Fidelity^TM^ DNA-polymerase using forward and reverse primers containing the restriction sites *Hin*dIII and *Bam*HI, respectively. The 4,656 bp PCR fragment was digested with both restriction enzymes and ligated with *Bam*HI/*Hin*dIII cleaved DNA of the high copy number *E. coli* cloning vector pUC18 (Amp^R^) (Norrander et al., 1983). After transformation of *E. coli* K12, a recombinant plasmid with the *scr* region was isolated from ampicillin-resistant transformants (data not shown).

**FIGURE 1:**
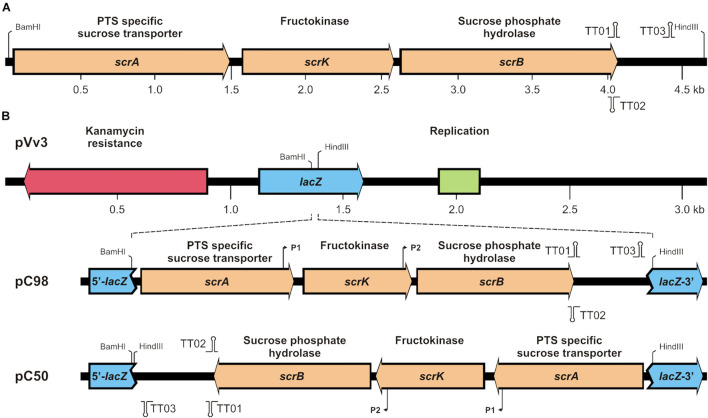
**(A)** Genetic map of the *scrA-K-B* region of strain *V. parahaemolyticus* 16-VB00198 and **(B)** integration of the cluster into shuttle vector pVv3. In **(B)** *lacZ* fragment containing a multiple cloning site the single restriction sites *Bam*HI and *Hin*dIII are shown in pVv3. In recombinant plasmids pC98 and pC50 putative promoters (P1, P2) and transcription terminators (TT01, TT02, TT03) are indicated.

The verified pUC18 (*scrAKB*) derivative was used for re-cloning of the 16-VB00198 *scrA-scrK-scrB* cluster into the *Vibrio/E. coli* shuttle vector pVv3 (Km^R^) (Klevanskaa et al., 2014). Finally, through selection of kanamycin-resistant *E. coli* K12 two recombinant plasmids derived from pVv3 were obtained. Both plasmids differed in the orientation of the inserted *scr* fragment with respect to the transcription direction of the *lacZ*α-fragment (Figure 1) and were further verified by commercial Sanger sequencing. In the recombinant plasmid pC50, the 30 bp *Bam*HI-*Hin*dIII fragment from the multiple cloning site of pVv3 is inserted on both sides of the *scr* fragment resulting in an opposite transcription direction of the *scr* genes and the *lacZ*α-fragment. In contrast, pC98 exhibited the same transcription direction of *scr* genes and *lacZ*α-fragment. Out of the literature (Blatch et al., 1990), two promoter and three transcription terminator sequences were deduced within the cloned fragment (Figure 1B), which are up to now not experimentally verified.

### Expression of the *scr* Gene Cluster in *Escherichia coli* and *Vibrio* spp.

**FIGURE 2:**
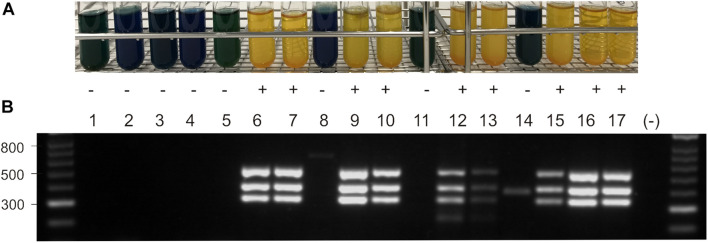
**(A)** Sucrose utilization of *E. coli* and *Vibrio* strains without plasmid, with vector pVv3, and with recombinant plasmids pC50 and pC98 in sucrose bouillon after growth at 37°C. Yellow color indicates growth (+) dark blue color no growth (–). **(B)** Agarose gel electrophorese of PCR products obtained with primers for *scrA, scrK* and *scrB* gene (331 bp, 410 bp, 527 bp; see Table 2). DNA ladder in bp, lane (–) shows PCR water control. Lane 1: *E. coli* K12, lane 2: BfR-VB00009, lane 3: BfR-VB00016, lane 4: BfR-VB00034, lane 5: *E. coli* K12 [pVv3], lane 6: *E. coli* K12 [pC50], lane 7: *E.coli* K12 [pC98], lane 8: BfR-VB00009 [pVv3], lane 9: BfR-VB00009 [pC50], lane 10: BfR-VB00009 [pC98], lane 11: BfR-VB00016 [pVv3], lane 12: BfR-VB00016 [pC50], lane 13: BfR-VB00016 [pC98], lane 14: BfR-VB00034 [pVv3], lane 15: BfR-VB00034 [pC50], lane 16: BfR-VB00034 [pC98], and lane 17: *V. parahaemolyticus* 16-VB00198.

*Escherichia coli* K12 strains are unable to utilize sucrose (Schmid et al., 1988). However, it was shown that after introduction of the *V. alginolyticus scr* gene cluster into *E. coli* K12, the clones are able to metabolize sucrose (Scholle et al., 1987). The expression of the *scr* genes of *V. parahaemolyticus* 16-VB00198 in *E. coli* K12 was tested in sucrose bouillon. While the original strain (GeneHogs^TM^ ) and its derivative carrying the vector pVv3 were negative for sucrose utilization, transformants harboring the recombinant plasmids pC50 or pC98 were capable of metabolizing the disaccharide (Figure 2A). To confirm the *E. coli* K12 transformants, a multiplex PCR targeting *scrA, scrB*, and *scrK* was applied using specific primers (Table 3). PCR amplification yielded PCR products of the expected sizes (Figure 2B).

To test if sucrose-negative *V. parahaemolyticus* and *V. vulnificus* can be converted to utilize the sugar, plasmids pC50 or pC98 were introduced into selected strains by electroporation using a protocol developed for pVv3 (Klevanskaa et al., 2014). Following transformation of three *Vibrio* strains (Table 1), kanamycin-resistant transformants were tested for sucrose utilization by growth in sucrose bouillon (Figure 2A). The transformants of the two *V. parahaemolyticus* strains (BfR-VB00009 and BfR-VB00016) and the *V. vulnificus* strain BfR-VB00034 were shown to be able to metabolize the sugar. The presence of the *scrABK* genes was also confirmed by *scr* multiplex PCR (Figure 2B).

### Presence of *scr* Cluster in Sucrose-Positive *Vibrio parahaemolyticus* Strains

To determine the occurrence of the *scr* gene cluster among *Vibrio* strains of our culture collection, a number of sucrose-positive *V. parahaemolyticus* strains was subjected to PCR application using the above described *scr* multiplex PCR. In total, 43 sucrose-positive strains, which had been found through biochemical testing were investigated for the presence of the *scr* genes. From all sucrose-positive strains the three expected PCR products were obtained confirming the presence of the *scr* DNA region (data not shown). Additionally, 10 sucrose-negative *V. parahaemolyticus* strains were tested by PCR, but did not yield any visible PCR products confirming that the PCR is specific for the detection of the *scr* gene cluster.

### Genetic Diversity of Sucrose-Positive *Vibrio parahaemolyticus* Strains

Whole-genome sequencing was performed to find out if the sucrose cluster is restricted to a small group of genetically related *V. parahaemolyticus* strains. In total, 17 sucrose-positive strains from our collection originating from different geographical origin, host animals and time point of isolation were selected for further dissection of their genetic basis. The resulting genomes of the strains were analyzed with respect to their phylogenetic relationship and their *scr* regions. WGS revealed that the genome sizes of the strains varied between 4.9 and 5.9 Mb (Supplementary Table S2). A MLST analysis of all 17 strains revealed that some strains belong to the same MLST sequence type (ST), however, several diverse alleles of housekeeping genes are found (see below).

**FIGURE 3:**
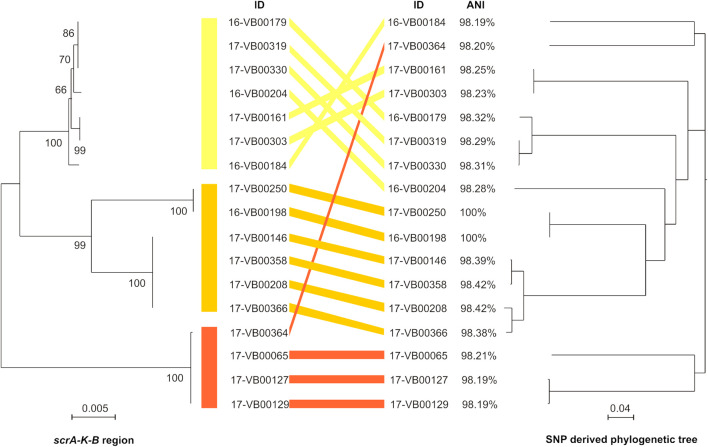
Comparison of sucrose *scrAKB* region (left half of figure) with an SNP-derived phylogeny tree (**right half of figure**) from 17 sucrose-positive *V. parahaemolyticus* strains. **Left half:** The evolutionary analyses for the *scrAKB* region was carried out by using the Maximum Likelihood method with Mega X (see section “Materials and Methods”). In the final dataset, 4,027 positions were included. The branch length is measured in number of substitutions per site and drawn to scale. Bootstrap values are shown at the nods. Right half: SNP tree was conducted using CSI Phylogeny 1.4 under default settings and the exclusion of heterozygous SNPs. Single nucleotide polymorphisms (SNPs) were called by mapping the genome of strain 16-VB00198 as reference. Scale bar represents the number of nucleotide substitutions per site. ANI value for each genome was calculated using the 16-VB00198 genome as reference.

The genomic region encompassing the coding sequences of the *scrA, scrK*, and *scrB* genes including the intergenic sequences (in total 4,027 bp) were analyzed using the Maximum Likelihood method. The sequences were assigned to three clusters, which are indicated by different colors (Figure 3, left half). The nucleotide identity of the *scr* regions of the strains ranged between 95.8 and 100%.

For determination of the phylogenetic relationship of the strains, the genome of 16-VB00198 was chosen as a reference for a SNP analysis. CSI phylogeny was used for SNP tree generation out of 17 sucrose-positive strains (see Figure 3, right half). In Supplementary Table S3, the SNP counts are displayed numerically. The percentage of the reference genome covered by all isolates is 73.59%, so 4,359,330 nucleotides represent the basis of the SNP comparison.

The SNP tree shows a distinct diversity between the sucrose-positive strains, as the SNP counts range between 7 (minimum) and 40,506 (maximum). The strains 16-VB00184, 16-VB00204, 17-VB00364, and 17-VB00065 differ between each other and all other strains by more than 35,000 SNPs. WGS also revealed that a number of strains with low differences in SNP counts between each other are closely related isolates. Such strains probably belong to the same yet unassigned multilocus sequence type (ST) according to the *V. parahaemolyticus* pubMLST scheme. In Supplementary Table S2, the alleles of housekeeping genes and their resulting STs are given. For unknown STs, the nearest alleles were given (Jolley et al., 2018).

As expected, the strains belonging to the same ST, are highly identical and exhibit *scrAKB* region only differing in a few nucleotides. In conclusion, the comparison of all genome sequences based on SNP differences reveals that the presence of sucrose utilizing genes is found in genetically diverse strains and is not restricted to a subset of closely related strains.

Additionally, the ANI values of all strains were calculated using the genome of 16-VB00198 as reference. The ANI values differ between 98.19 and 98.42% when compared to the reference. The genome of strain 17-VB00250 is 100% identical to the reference genome, corresponding to the low the difference in SNPs (only seven SNPs). The ANI values are displayed in Figure 3, however, their distinctiveness is less than that of the SNP analysis.

### Integration Site of *scr* Gene Cluster Within the *Vibrio parahaemolyticus* Genome

**FIGURE 4:**
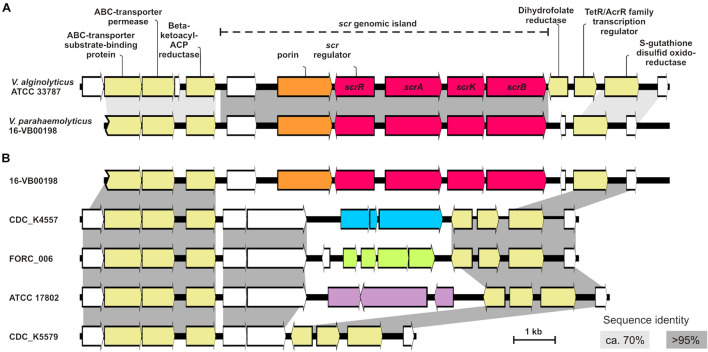
**(A)** Comparison of the genetic region with the sucrose metabolizing genes (*scr* island) of strain *V. parahaemolyticus* 16-VB00198 to the corresponding region of *V. alginolyticus* ATCC 33787 (accession CP013485). *scr* genes are colored red, porin-coding genes in orange, hypothetical genes are in white, and annotated genes are in yellow. **(B)** Comparison of the genetic region of chromosome II of 16-VB00198 to the corresponding region of sucrose-negative *V. parahaemolyticus* strains (CDC-K4557 accession CP006007, strain FORC-006 accession CP009766, strain ATCC 17802 accession LATW01000005, strain CDC_K5579 accession MIVB01000118). Gene clusters in blue, in green and in purple indicate annotated genes with different functions. Sequence identity is shown in light gray (approximately 70%) and in dark gray (>95%).

We compared the *scrAKB* region of strain *V. parahaemolyticus* 16-VB00198 to the genome of the *V. alginolyticus* reference strain ATCC 33787 (accession CP013485). In general, these two Vibrio species are related belonging to the *Vibrio harveyi* clade (Ke et al., 2018) and sucrose utilization has been used as an important phenotypic feature for biochemical differentiation of these bacteria. The comparison of the *scr* region of the two *Vibrio* strain genomes is depicted in Figure 4A. The cloned region is part of a genomic island, which may have been acquired through horizontal gene transfer from *V. alginolyticus*. In this species all strains are sucrose-positive (Farmer et al., 2015), whereas in *V. parahaemolyticus* 99% of the strains are unable to utilize sucrose. In *V. parahaemolyticus* 16-VB00198, the sucrose genes *scrA, scrK, and scrB* are part of a genomic island of approximately 8.0 kb. The island has an overall nucleotide similarity of >95% to the region of *V. alginolyticus*. Interestingly, the location of the *scr* gene cluster was determined to be at the same position in chromosome II of *V. alginolyticus* and *V. parahaemolyticus*. In the flanking sequences of the *scr* genomic island the similarity between the chromosomal DNA sequences of the two species drops to approximately 70%. The identified scr island contains additional coding sequences (CDS) in both species: *scrR*, a gene coding for a putative transcriptional regulator upstream of *scrA*, a gene encoding a porin protein possibly involved in disaccharide uptake and a CDS of a hypothetical protein. The direction of transcription of all island genes is depicted in Figure 4A. Downstream of the *scrB* gene, the genomic island ends in a short pseudogene sequence (110 bp), which is derived from a gene encoding a dihydrofolate reductase. On the 5′ end of the *scr* genomic island the same genes encoding metabolic enzymes are present in both strains (immediately upstream of the island is a gene for a ß-ketoacyl-ACP reductase). On the 3′ end of the *V. parahaemolyticus* island the first common gene with a chromosomal gene of *V. alginolyticus* is a gene coding for a *S*-glutathione transferase. Two genes present in *V. alginolyticus* ATCC 33787 between the scrB gene of the genomic island and the S-glutathione transferase gene are absent in *V. parahaemolyticus* 16-VB00198. In the 17 genomes of the sucrose-positive *V. parahaemolyticus* strains, the same arrangement of genes is conserved. The overall nucleotide identity of the *scr* genomic island and its flanking chromosomal region among the *V. parahaemolyticus* strains is higher than 97% (14 kb of contig_16 of 16-VB00198 from nucleotide position 25,001–40,000). The GC contents of the 8 kb region of the *scr* island of each *V. parahaemolyticus* genome (start codon of the hypothetical gene to the stop codon of *scrB* gene) was calculated and compared to the GC contents of the corresponding whole genome. In all 17 cases the GC contents of the *scr* island is 1.5% lower than that of the whole genome (see Supplementary Table S2). This result supports the hypothesis of acquisition of the *scr* island through horizontal gene transfer.

We also performed BlastN searches with the chromosomal *scr* region of *V. alginolyticus* ATCC 33878 (accession CP013485; nucleotide position 1,012,100 to 1,027,000) against other published *V. alginolyticus* genomes. In some *V. alginolyticus* strains, the region contains the same number and identical arrangement of genes as depicted in Figure 4A. However, in various other *V. alginolyticus* strains the two genes between *scrB* and the *S*-glutathione transferase gene are missing (just as with the sucrose-positive *V. parahaemolyticus* strains, data not shown).

We were furthermore interested which genes are present in the corresponding chromosome II region of sucrose-negative *V. parahaemolyticus* strains (Figure 4B). This genomic region displays a greater genetic variability between the strains. Downstream of the gene encoding ß-ketoacyl-ACP reductase two conserved genes encoding proteins of unknow function are common in the analyzed strains (indicated by dark gray boxes, Figure 4B). Downstream of these two hypothetical protein CDS, more genetic variability is found. Four examples of genomes of sucrose-negative strains are shown in Figure 4B: In strains CDC-K4557, FORC-006, and ATCC 17802 different gene clusters follow (see accession for details), whereas in strain CDC_K5579 nothing more than the two common hypothetical proteins CDS are present. In all genomes of sucrose-negative strains two genes for the dihydrofolate reductase gene and the transcription regulator are present upstream of the *S*-glutathione transferase gene as found in the genome of *V. alginolyticus* ATCC 33878 (Figure 4B).

## Discussion

The aim of our study was to characterize the genetic background for sucrose utilization in sucrose-positive strains of the species *V. parahaemolyticus*, of which most strains are unable to metabolize sucrose as carbon source. By BlastN comparison with known genes of the related species *V. alginolyticus* (Scholle et al., 1987, 1989; Blatch et al., 1990; Blatch and Woods, 1991) the genes *scrA* (encoding a sucrose uptake protein), *scrK* (coding for a fructokinase) and *scrB* (coding for a sucrose-6-phosphate hydrolase) were identified. The proteins of these genes degrade the disaccharide to phosphorylated sugars (fructose-6-phosphate and glucose-6-phosphate) which enter the glycolytic pathway (Reid and Abratt, 2005). The *scrA* gene product is a sucrose-specific protein of the phosphoenolpyruvate dependent phosphotransferase system (PTS) which transports sucrose through the inner membrane by phosphorylation of the disaccharide (Reid and Abratt, 2005; Deutscher et al., 2006).

We inserted the three genes into the *Vibrio/E. coli* shuttle vector pVv3 (Klevanskaa et al., 2014) and constructed two recombinant plasmids pC50 and pC98 containing the gene cluster in different orientations. Usually *E. coli* K12 strains are unable to ferment sucrose (Schmid et al., 1988). However, the derivative strains carrying pC50 or pC98 grew in sucrose bouillon. A similar result was reported for the *V. alginolyticus scrAKB* genes (Scholle et al., 1987) when introduced into *E. coli* K12. After transformation of the recombinant plasmid into sucrose-negative *V. parahaemolyticus* and *V. vulnificus* strains, the transformants were able to utilize the disaccharide. In the three species, the expression of the *scr* genes was independent of their transcription orientation in respect to the *lacZ*α-fragment suggesting that the expression is driven by promoters within the pVv3 plasmid. Thus, we demonstrated that these three enzymes are sufficient for the metabolization of the disaccharide for catabolic pathways. By performing a multiplex PCR targeting *scrA, scrK*, and *scrB* we confirmed that sucrose utilizing *V. parahaemolyticus* strains harbor this gene cluster, as all 43 sucrose-positive isolates from our strain collection were positive. The sequence similarity between all *scr* clusters was higher than 95.8%. Whole genome sequencing confirmed that several strains are genetically highly diverse (>35,000 SNPs) and do not belong to a genetically related subset of strains.

In two recent publications the sucrose utilization in *Vibrio* and the related species *Photobacterium* was addressed. One paper describes the presence of a sucrose utilization cluster in a sucrose utilizing *V. parahaemolyticus* isolate from a shrimps aquaculture (De Mesa et al., 2021). The strain was subjected to WGS and in a bioinformatic approach the genome was compared to that of a sucrose-negative strain. The authors identified the same arrangement of sucrose utilizing genes in the sucrose-positive strain, as determined in this study (De Mesa et al., 2021). Upstream of the scrA-K-B region in the analyzed *V. parahaemolyticus* strain also a *scrR* gene and a porin gene are present. The putative *scrR* gene encodes a LacI-GalR family transcription regulator controlling expression of the *scrB* gene in response to sucrose (Reid and Abratt, 2005). Upstream of *scrR* a gene encoding a porin is located in all strains. This porin belongs to the LamB family whose members are able to bind various sugars and to transport them through the outer membrane. A related porin is the *Salmonella typhimurium* sucrose-specific porin ScrY (Reid and Abratt, 2005). In our genetic constructs, the porin gene of the *scr* island of *V. parahaemolyticus* was not included. However, the metabolization of sucrose in transformants of *V. parahaemolyticus* and *V. vulnificus* show that other porins in these *Vibrio* strains can transport the disaccharide through the outer membrane.

The arrangement of *scr* genes in *Photobacterium damselae*, a marine species belonging to the family *Vibrionaceae*, is also very similar to that of *V. alginolyticus* and *V. parahaemolyticus* (Abushattal et al., 2020). The authors performed a bioinformatic analysis for *scr* genes in several published genomes of *Vibrio* and *Photobacterium* species which revealed the arrangement of *scrA, scrK*, and *scrB* with the putative regulator gene *scrR* upstream of *scrA* in many species of these two genera (Abushattal et al., 2020). The *V. alginolyticus* sucrose uptake and utilization system represents the prototype for the metabolization of this disaccharide in many species of the *Vibrionaceae*.

In *Photobacterium damselae* two direct repeat sequences flanking the four *scr* genes were identified and it was hypothesized that these sequences may be involved in horizontal gene transfer (Abushattal et al., 2020). We could not find repeat sequences flanking the *scr* genomic cluster in our *V. parahaemolyticus* genomes. All 17 *scr* clusters were integrated in the same site between genes, which are located in chromosome II. For this reason, a plasmid borne origin of the *scr* genomic island is in our investigated sucrose-positive stains highly unlikely. Horizontal gene transfer (HGT) is a widely used mechanism in which bacteria exchange genetic material. The close similarity of the *scr* genomic islands between *V. alginolyticus* and *V. parahaemolyticus* suggests that this genetic structure was acquired by HGT. This hypothesis is also supported by comparison of the GC contents of the *scr* islands to the GC contents of the whole genomes in all 17 sucrose-positive *V. parahaemolyticus* strains. The GC content of the *scr* islands is 1.5% lower than that of the complete genome in all strains.

In conclusion, we characterized a genomic island within *V. parahaemolyticus* strains, which carries genes encoding proteins for uptake and degradation of sucrose. We could verify the function of three genes *scrA, scrK*, and *scrB* by cloning and expression in *V. parahaemolyticus* and *V. vulnificus*. Recombinant pVv3 plasmids with the *scr* genomic island were also functional in *E. coli* K12. A multiplex PCR targeting the three *scr* genes showed that this gene cluster is present in sucrose-positive *V. parahaemolyticus* strains. By WGS of 17 sucrose-positive *V. parahaemolyticus* we could show that the genes are arranged in a conserved genomic island which is closely related to the *scr* genomic region of *V. alginolyticus*. The island is found in genetically diverse strains and not restricted to a small subgroup of *V. parahaemolyticus* strains. Food laboratories testing biochemical phenotypes could use the multiplex PCR targeting the *scrAKB* genes if sucrose-positive *V. parahaemolyticus* isolates are detected.

## Data Availability Statement

This Whole Genome Shotgun project has been deposited at DDBJ/ENA/GenBank under the accession numbers given in Supplementary Table S2.

## Author Contributions

ES and JH designed the study. CG, CJ, FS, and HG performed the experiments. JH, CG, and ES analyzed the data. ES and JH prepared the tables and figures. All the authors edited the manuscript.

## Conflict of Interest

The authors declare that the research was conducted in the absence of any commercial or financial relationships that could be construed as a potential conflict of interest.

## Publisher’s Note

All claims expressed in this article are solely those of the authors and do not necessarily represent those of their affiliated organizations, or those of the publisher, the editors and the reviewers. Any product that may be evaluated in this article, or claim that may be made by its manufacturer, is not guaranteed or endorsed by the publisher.

## Footnotes

1
enve-omics.ce.gatech.edu/ani/

2
pubmlst.org/organisms?title=vibrio+parahemolyticus

3
cge.cbs.dtu.dk/services/PlasmidFinder/

4
cge.cbs.dtu.dk/services/CSIPhylogeny/
