# Supplementary material for: The Acquisition of the scr Gene Cluster Encoding Sucrose Metabolization Enzymes Enables Strains of Vibrio parahaemolyticus and Vibrio vulnificus to Utilize Sucrose as Carbon Source
Source: Front Microbiol. 2021 Nov 15;12:754464. doi: 10.3389/fmicb.2021.754464 (PMC8636126; doi:10.3389/fmicb.2021.754464)
Supplement: Supplementary file 1 [file Table_1.DOCX]

Supplementary Material

# Supplementary Data

**Table S1** Primers used for sequencing of *scr* cluster.

| **Primer** | **Sequence 5´-3´** |
| --- | --- |
| M13 puc-Fo* | CCC AGT CAC GAC GTT GTA AAA CG |
| M13 puc-Re* | AGC GGA TAA CAA TTT CAC ACA GG |
| scrSe1 | TGC GAT TGA AAC GCA ACT TC |
| scrSe2 | ACT GGC AAA TGA GCC AAG TC |
| scrSe3 | ATC GCC GAC TTG AGC AAA TC |
| scrSe4 | AAA TCG ACA CAC CAT CGG TC |
| scrSe5 | AGC ATC TCG AAC CTA ACT CAC |
| scrSe6 | AAT GAG TAA GCC CAA CTC GG |
| scrSe7 | TAG GAC CTT TAT GCT GCC AC |
| scrSe8 | AAT TCT GTC TGG GTC GGA AG |
| scrSe9 | AAA GCC CTG ATA GTC CAA CG |
| scrSe10 | CGT CAA CTT CGC CAT TTC TG |

*Primers used in recombinant plasmids pC98 and pC50
